# Prenatal tobacco and alcohol exposure, white matter microstructure, and early language skills in toddlers from a South African birth cohort

**DOI:** 10.3389/fnint.2024.1438888

**Published:** 2024-09-02

**Authors:** Chloe Scholten, Mohammad Ghasoub, Bryce Geeraert, Shantanu Joshi, Catherine J. Wedderburn, Annerine Roos, Sivenesi Subramoney, Nadia Hoffman, Katherine Narr, Roger Woods, Heather J. Zar, Dan J. Stein, Kirsten Donald, Catherine Lebel

**Affiliations:** ^1^Hotchkiss Brain Institute, University of Calgary, Calgary, AB, Canada; ^2^Alberta Children's Hospital Research Institute, University of Calgary, Calgary, AB, Canada; ^3^Department of Neurology, Ahmanson-Lovelace Brain Mapping Center, University of California, Los Angeles, Los Angeles, CA, United States; ^4^Department of Bioengineering, University of California, Los Angeles, Los Angeles, CA, United States; ^5^Division of Developmental Paediatrics, Department of Paediatrics and Child Health, Red Cross Memorial Children's Hospital, University of Cape Town, Cape Town, South Africa; ^6^Neuroscience Institute, University of Cape Town, Cape Town, South Africa; ^7^Department of Psychiatry and Mental Health, University of Cape Town, Cape Town, South Africa; ^8^South African Medical Research Council (SAMRC), Unit of Risk and Resilience in Mental Disorders, University of Cape Town, Cape Town, South Africa; ^9^Department of Psychiatry and Biobehavioural Sciences, University of California, Los Angeles, Los Angeles, CA, United States; ^10^The Semel Institute for Neuroscience and Human Behaviour, University of California, Los Angeles, Los Angeles, CA, United States; ^11^David Geffen School of Medicine, University of California, Los Angeles, Los Angeles, CA, United States; ^12^Department of Paediatrics and Child Health, Red Cross War Memorial Children's Hospital, University of Cape Town, Cape Town, South Africa; ^13^South African Medical Research Council (SAMRC), Unit on Child and Adolescent Health, University of Cape Town, Cape Town, South Africa; ^14^Department of Radiology, University of Calgary, Calgary, AB, Canada

**Keywords:** diffusion tensor imaging (DTI), early language, prenatal tobacco exposure, prenatal alcohol exposure (PAE), development

## Abstract

**Introduction:**

Tobacco and alcohol are the two most common substances used during pregnancy, and both can disrupt neurodevelopment, resulting in cognitive and behavioral deficits including language difficulties. Previous studies show that children with prenatal substance exposure exhibit microstructural alterations in major white matter pathways, though few studies have investigated the impact of prenatal substance exposure on white matter microstructure and language skills during the toddler years.

**Methods:**

In this study, 93 children (34 exposed to alcohol and/or tobacco) aged 23 years from the Drakenstein Child Health Study, South Africa, completed Expressive and Receptive Communication assessments from the Bayley Scales of Infant and Toddler Development, Third Edition (BSID-III) and underwent diffusion MRI scans. Diffusion images were preprocessed, and 11 major white matter tracts were isolated. Fractional anisotropy (FA) and mean diffusivity (MD) were extracted for each white matter tract. Linear regression was used to examine differences between the tobacco/alcohol exposed group and unexposed controls for FA, MD, and language scores, as well as relationships between brain metrics and language. There were no significant group differences in language scores or FA.

**Results:**

Children with alcohol or tobacco exposure had lower average MD in the splenium of the corpus callosum compared to unexposed controls. Significant interactions between prenatal substance exposure and language scores were seen in 7 tracts but did not survive multiple comparisons correction.

**Discussion:**

Our findings show that prenatal alcohol and/or tobacco exposure appear to alter the relationship between white matter microstructure and early language skills in this population of toddlers, potentially laying the basis of language deficits observed later in older children with prenatal substance exposure, which may have implications for learning and interventions.

## 1 Introduction

Alcohol and tobacco consumption are common in pregnancy, and both substances can cross the placenta and exert maladaptive effects on the developing fetus (Behnke and Eyler, 1993; Veazey et al., 2013; Caputo et al., 2016). It is estimated that upwards of ~10% of pregnant individuals consume alcohol and 1.7% consume tobacco worldwide (Lange et al., 2018; Popova et al., 2023). This prevalence is higher in some countries like South Africa, where ~20% of people report using alcohol (Petersen Williams et al., 2014), and ~5% use tobacco (Phaswana-Mafuya et al., 2019) during pregnancy in some regions.

Alcohol is a teratogen that can disrupt neuronal proliferation and migration and can cause cell death in the fetal brain (Miller, 1986; Veazey et al., 2013). In addition to its direct effects on fetal brain development, alcohol causes decreased umbilical artery blood flow which induces hypoxia in the fetus (Jones et al., 1981; Mukherjee and Hodgen, 1982; Bosco and Diaz, 2012) and can lead to growth retardation (Abel, 1984, 1985; Lo et al., 2017). As a result, individuals with prenatal alcohol exposure (PAE) may demonstrate cognitive and behavioral deficits (Lees et al., 2020; Popova et al., 2023) as well as neuroanatomical changes such as reduced gray matter and white matter volume and altered white matter microstructure (Colby et al., 2012; Donald et al., 2015; Kar et al., 2021; Moore and Xia, 2022).

Maternal tobacco use during pregnancy can also result in impaired fetal growth, and reduced gestation time leading to premature birth (Ward et al., 2007). Nicotine reduces the availability of nutrients and oxygen to the fetus by acting as a powerful vasoconstrictor (Espy et al., 2011). Additionally, neurodevelopment is disrupted when nicotine (rather than endogenous acetylcholine) binds to nicotinic acetylcholine receptors (nAChRs) during gestation (Navarro et al., 1989; Heath and Picciotto, 2009). Studies have also shown lower total cortical volume and surface area (Gonzalez et al., 2023) and white matter alterations in the uncinate fasciculus, corticospinal tract, and the genu of the corpus callosum (Jacobsen et al., 2007; Roos et al., 2021) for children with prenatal tobacco exposure (PTE). PTE has been associated with decreased cognitive performance in children (Ernst et al., 2001; Margolis et al., 2021) and reduced language abilities (Hernández-Martínez et al., 2017). PAE and PTE frequently co-occur (Dukes et al., 2017).

Evidence is mixed when investigating the extent to which PAE or PTE affect language development (Peixinho et al., 2022). Several studies demonstrate that moderate or heavy PAE impairs language skills in early childhood (Fried and Watkinson, 1990; Fried et al., 1992; Hendricks et al., 2019), though others show no major deficits (Hendricks et al., 2020; Lindinger et al., 2022). Longitudinal studies indicate language difficulties at 2-6 years of age in children with PAE and PTE, but no difference when the children were 4-6 years old (Fried and Watkinson, 1990; Fried et al., 1992). Few studies have investigated language skills in PTE. Additionally, a cohort study by Hernández-Martínez et al., 2017 found that infants with PTE had impaired language scores related to auditory function including vocalizations, sound discrimination, and prelinguistic vocalizations. The lack of clarity in previous studies highlights the importance of studying the effects of PAE and/or PTE (collectively termed prenatal substance exposure, PSE) in early childhood, as well as the underlying neural mechanisms of language development.

Magnetic resonance imaging (MRI) offers the capability to non-invasively examine brain structure (Yen et al., 2023). Moreover, white matter microstructure specifically can be assessed using diffusion imaging (dMRI) (Mürner-Lavanchy et al., 2018). Fractional anisotropy (FA) and mean diffusivity (MD) are metrics that are sensitive to microstructural properties including local fiber density, coherence, orientation, and myelination (Basser and Jones, 2002; Beaulieu, 2002). Previous literature has established a well-recognized relationship between white matter microstructure and early language skills (Mills et al., 2013; Mürner-Lavanchy et al., 2018; Walton et al., 2018), reflected by positive relationships between language/reading abilities and FA, and negative relationships with MD in major white matter tracts including the arcuate fasciculus, uncinate fasciculus, inferior fronto-occipital fasciculus, and the corpus callosum (Vandermosten et al., 2012, 2015; Saygin et al., 2013; Walton et al., 2018; Sket et al., 2019; Zuk et al., 2021).

Few studies have examined how PSE affects the relationship between white matter microstructure and language abilities, focusing only on PAE (Gómez et al., 2022; Ostertag et al., 2023). Gómez et al. (2022) found negative associations between speeded naming and FA in the left superior longitudinal fasciculus and left inferior longitudinal fasciculus in 7-16 year olds with PAE. Ostertag et al. (2023) reported altered developmental trajectories in the arcuate fasciculus of preschool aged children with PAE, and a significant FA-by-age-by-group interaction for the left arcuate in predicting speeded naming scores, and right arcuate FA predicting phonological processing scores (Ostertag et al., 2023). Treit et al. (2013) reported significant associations between changes in MD in the superior fronto-occipital fasciculus and superior longitudinal fasciculus, with changes in reading abilities over time in children aged 5-13 years. The relationship between early language skills and diffusion metrics in toddlers with PAE remains unknown.

It is important to understand how PSE affects white matter microstructure and early language skills in toddlers to inform early interventions and mitigate difficulties as the children get older. The current study aimed to investigate white matter microstructure and language, and the relationships between them, in a cohort of South African toddlers with PSE (defined as PAE, PTE, or both).

## 2 Methods

### 2.1 Participants

Ninety three children aged 2–3 years (58 males, mean = 2.77 +/−0.14 years) were included in this study. In total, nine children had PAE (but not PTE), 11 children had PTE (and no PAE), 14 children had both PAE and PTE, and 59 children had neither PTE nor PAE (Table 1). Children were part of the larger Drakenstein Child Health Study (DCHS); a multidisciplinary longitudinal birth cohort study in Western Cape, South Africa (Stein et al., 2015; Donald et al., 2018). The DCHS aims to investigate the early-life determinants of infant and child development and cognition (Donald et al., 2018). The Western Cape is culturally and linguistically heterogenous and characterized by low socioeconomic status (Donald et al., 2018). A total of 1143 children were included in DCHS. 121 children were recruited for MRI scans at 2–3 years. Of those, n = 28 did not provide high quality data for analysis. Informed parent/guardian consent was obtained. The DCHS was approved by the University of Cape Town Human Research Ethics Committee (401/2009). The neuroimaging sub-study was approved by the University of Cape Town Human Research Ethics Committee (UCT HREC, Reference 525/2012).

**Table 1:** Participant demographics and language scores. PSE: children with PAE and/or PTE. Control: unexposed children.

	**PSE (*n* = 34)**	**Control (*n* = 59)**	***t*-test *P*-value**
Exposure(s)	PAE Only: 9 PTE Only: 11 Both: 14	-	-
Age (months)	32.40	33.49	0.032^*^
Sex	M = 22 (64.7%)	M = 36 (61.0%)	0.895
Household Income (monthly)	< R1000/m” = 11 R1000–5000/m” = 19 >R5000/m” = 4	< R1000/m” = 11 R1000–5000/m” = 39 > R5000/m” = 9	0.322
Expressive communication score (*n* = 80)	6.733	7.294	0.413
Receptive communication score (*n* = 83)	6.625	6.925	0.561

^*^Indicates statistically significant (p > 0.05); 1 South African Rand (R) = ~0.054 United States Dollar (USD).

PSE was evaluated using parental questionnaires as well as the Alcohol, Smoking and Substance Involvement Screening Test (ASSIST), a tool developed by the World Health Organization to screen for substance use in adults (WHO ASSIST Working Group, 2002) and has been validated in previous studies (Humeniuk et al., 2008; Roos et al., 2021). Twenty three children had confirmed PAE, with a minimum score of 11 on ASSIST; six children had low exposure, 10 had moderate exposure, five had high exposure, and 2 had no ASSIST scores, but PAE was confirmed by parents. Twenty five participants had PTE, 17 of which had moderate exposure (ASSIST score ≥ 6) and 8 had high exposure (ASSIST score ≥ 28); no children had low exposure to PTE. Children with confirmed prenatal exposure to substances other than alcohol and tobacco (e.g., cannabis, cocaine) were excluded. Additional exclusion criteria included children who: were born < 36 weeks gestation, had genetic and/or congenital disorders, or had contraindications to MRI. Children spoke English, Afrikaans, Shona, or isiXhosa.

### 2.2 Language assessments

Trained assessors administered the Expressive and Receptive Communication subtests from the Bayley Scales of Infant and Toddler Development, Third Edition (BSID-III; Bayley, 2012) to assess child early language and communication skills. Expressive Communication assesses skills such as gesturing and naming objects and pictures. Receptive Communication assesses the child's ability to recognize and identify sounds, objects, and pictures. Assessments were translated from English (forward and back translation) and completed in the participant's preferred language.

### 2.3 Image acquisition

Children underwent diffusion MRI in a 3T Siemens Skyra MRI scanner with a 32-channel head coil at Groote Schuur Hospital in Cape Town, South Africa. DTI data was acquired using voxel size = 1.8 × 1.8 × 2.0 mm, TR = 7,800 ms; TE = 92 ms, with 30 gradient diffusion directions at b = 1,000 s/mm^2^, and one b = 0 s/mm^2^; one full protocol was acquired in each of the anterior-posterior and posterior-anterior phase encoding directions. All scans were performed during natural sleep. Further details about the MRI image acquisition are described in Wedderburn et al. (2020).

### 2.4 Image processing

**Figure 1:**
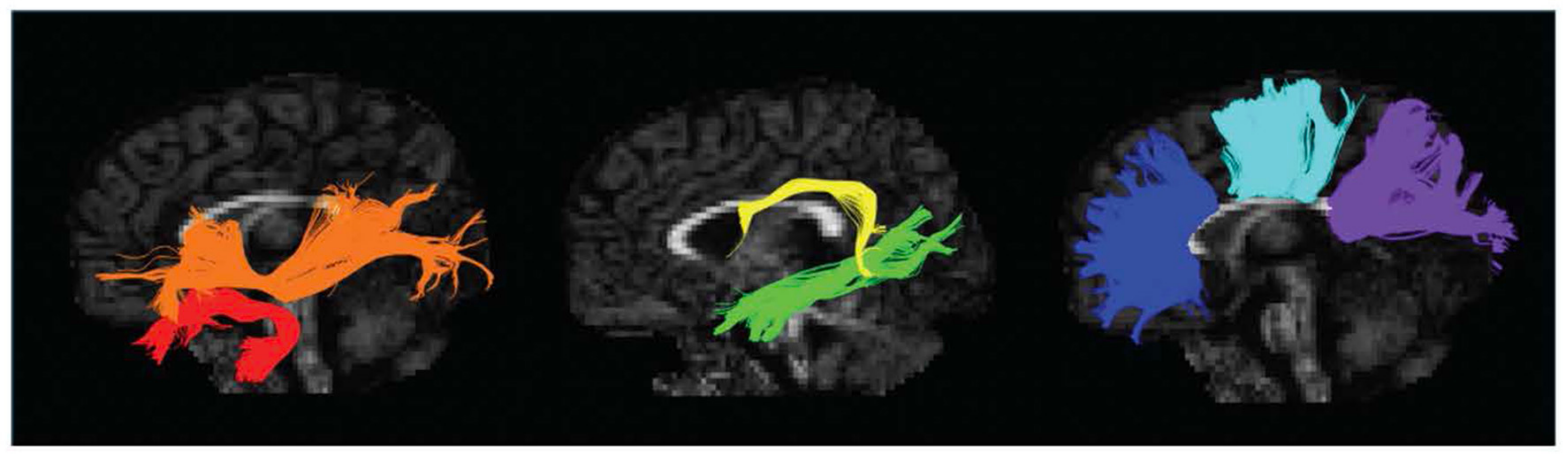
Left sagittal view of isolated white matter tracts. Red, Uncinate Fasciculus (UF); Orange, Inferior Fronto-Occipital Fasciculus (IFOF); Yellow, Arcuate Fasciculus (AF); Green, Inferior Longitudinal Fasciculus (ILF); Blue, Genu of the Corpus Callosum (CC); Aqua, Body of the Corpus Callosum (CC); Purple, Splenium of the Corpus Callosum (CC).

DTI data was preprocessed using ExploreDTI, a graphical toolbox for processing, analyzing, and visualizing MR data that is written in MATLAB (Leemans et al., 2009; MATLAB version: 9.6.0.1472908, The MathWorks Inc, 2022). Our image preprocessing including correction for Gibbs ringing, subject motion, eddy current, and EPI-based susceptibility distortions. The diffusion tensor was calculated via the REKINDLE method (Tax et al., 2015), and whole brain tractography was performed with the following parameters: seedpoint resolution = 2 × 2 × 2 mm^3^, FA threshold = 0.15, fiber length = 50–500 mm, angle threshold = 30°, step size = 1 mm. Semiautomated tractography was used to delineate 11 major white matter tracts associated with language processing: the bilateral longitudinal fasciculus (ILF), bilateral uncinate fasciculus (UF), bilateral inferior fronto-occipital fasciculus (IFOF), bilateral arcuate fasciculus (AF), and three components of the corpus callosum (CC)—genu, body, and splenium (Figure 1) (Smits et al., 2014; Kargar and Jalilian, 2024). Mean FA and MD were extracted for each tract in each individual.

Two prior studies examined brain diffusion properties in an overlapping sample of participants using tract-based spatial statistics (TBSS). Here, we chose to use tractography so we could focus our analysis on specific tracts a priori, analyze those tracts as a whole, and because tractography is less sensitive to small errors in registration across participants than TBSS.

### 2.5 Statistical analysis

All statistical analysis was completed using R studio (v4.2.1, R Core Team, 2021). Expressive and Receptive Communication scores, age, and FA and MD of each tract were compared between groups using t-tests. Sex differences between groups were assessed using a chi-squared test. Linear regression models were used to test associations between white matter microstructure (FA, MD) and early language skills, as well as the extent to which PSE moderates these associations. Receptive or Expressive Communication scores were the dependent variable, with age, sex, median household income (average household income per month), FA or MD (one at a time), PSE, and the interaction term between DTI metrics and PSE as independent variables, according to the following:

Receptive/Expressive Communication ~ age + sex + household income + FA/MD + FA/MD ^*^ PSE.

A total of 44 models were tested: 11 white matter tracts x two diffusion metrics (FA, MD) x two language scores (Receptive Communication, Expressive Communication).

Child's primary language was considered as an additional covariate, but no significant correlations were observed with communication scores during preliminary analysis, so this factor was excluded from subsequent models. False discovery rate (FDR) was implemented to correct for multiple comparisons at *q* < 0.05. FDR correction was completed separately for Expressive and Receptive Communication models. A total of 22 comparisons were made using FDR for each effect (ex: household income, PSE, PSE x brain interaction).

*Post hoc* analyses were performed to examine the effects of PAE and PTE separately, compared to controls with no exposure to either substance. The same linear regression models were used for PAE and PTE as were used for PSE.

Given the known relationship between cognition and language, we also investigated whether the two groups had different cognitive scores on the BSID-III Cognitive Scale, and if PSE influenced the relationship between white matter microstructure and Cognitive Scores.

## 3 Results

### 3.1 Language scores

An independent samples t-test showed no significant differences in Expressive (*p* = 0.41) or Receptive (*p* = 0.56). Communication scores between the PSE and control groups (Table 1).

### 3.2 White matter microstructure

**Figure 2:**
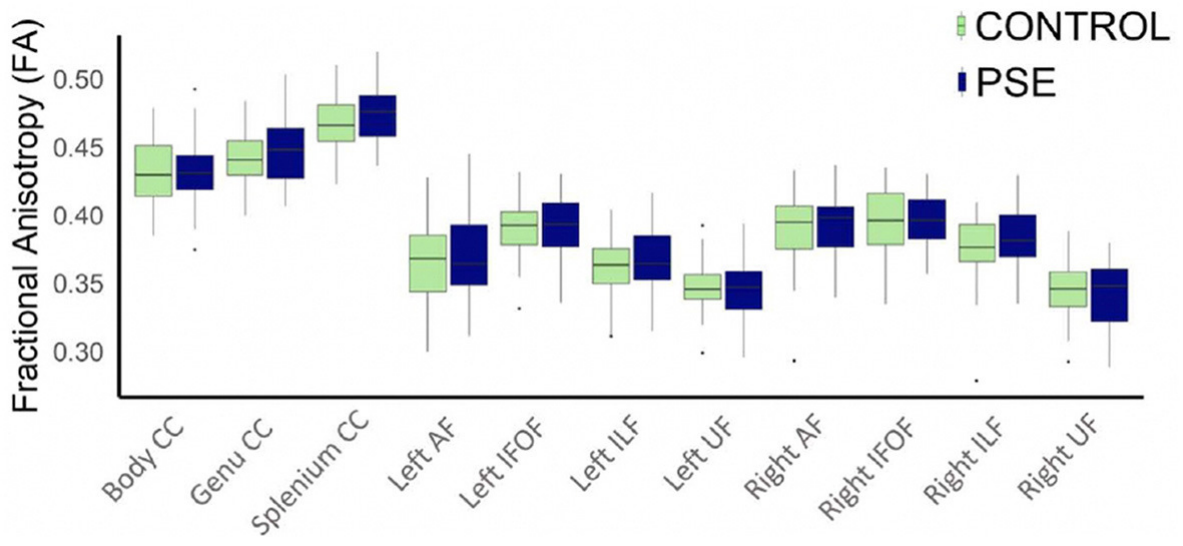
Boxplot displaying mean FA values of the white matter tracts for prenatal substance exposure (PSE; defined as prenatal alcohol and/or tobacco exposure) and control groups. FA values were directly pulled from the white matter for each participant interest variables have not been regressed out. No group differences in FA were significant. ILF, inferior longitudinal fasciculus; UF, uncinate fasciculus; IFOF, inferior fronto-occipital fasciculus; AF, arcuate fasciculus; CC, corpus callosum.

**Figure 3:**
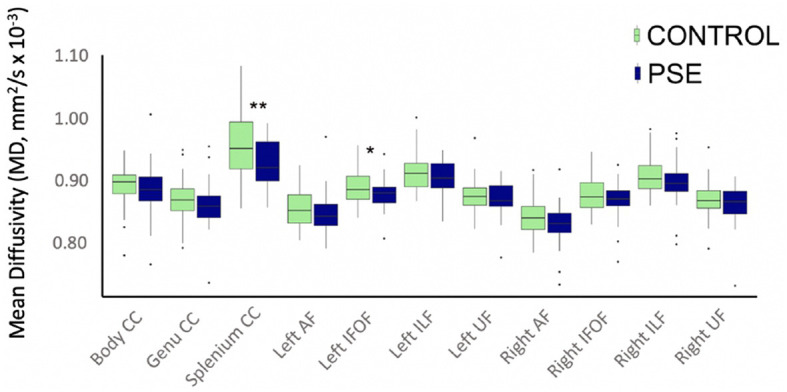
Boxplot displaying mean MD values of the white matter tracts for prenatal substance exposure (PSE; defined as prenatal alcohol and/or tobacco exposure) and control groups. MD values were directly pulled from the white matter for each participant interest variables have not been regressed out. MD was significantly lower in the PSE group compared to controls in the left IFOF (p = 0.030; q = 0.33) and the splenium of the CC (p = 0.002, q = 0.034). ILF, inferior longitudinal fasciculus; UF, uncinate fasciculus; IFOF, inferior fronto-occipital fasciculus; AF, arcuate fasciculus; CC, corpus callosum.

The arcuate fasciculus could not be delineated in every individual, so the right AF had 89 data points and the left AF had 83 data points. All other tracts were delineated in all individuals. There were no significant group differences of FA in any white matter tract (Figure 2). The PSE group had lower MD in the left IFOF (*p* = 0.030) and the splenium of the CC (*p* = 0.002) (Figure 3). After FDR corrections, only the differences in the splenium remained significant (*q* = 0.034).

### 3.3 Substance exposure

**Figure 4:**
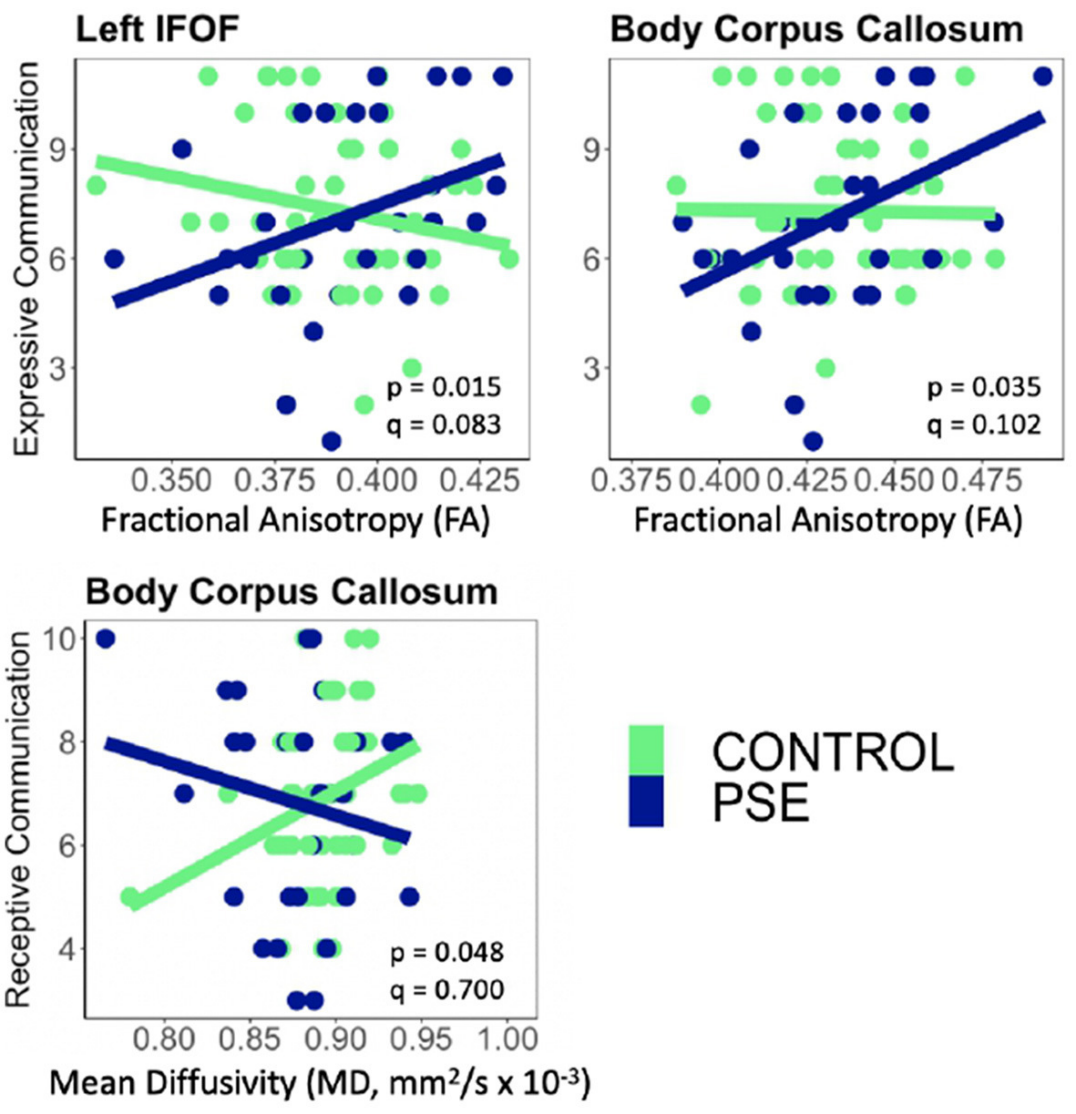
There were significant interactions between Prenatal Substance Exposure (PSE; defined as prenatal alcohol and/or tobacco exposure) and FA for Expressive Communication scores in the left IFOF and body of the CC. PSE had an interaction with MD in the body of the CC for Receptive Communication scores. Interaction effects did not survive multiple comparisons correction (*q* > 0.05 for all). The top row of plots displays FAxPSE interactions for Expressive Communication while the bottom row shows MDxPSE interactions for Receptive Communication. PSE group (*n* = 34) can be visualized in blue, and the control group (*n* = 59) is shown in green. IFOF, Inferior Fronto-Occipital Fasciculus.

**Figure 5:**
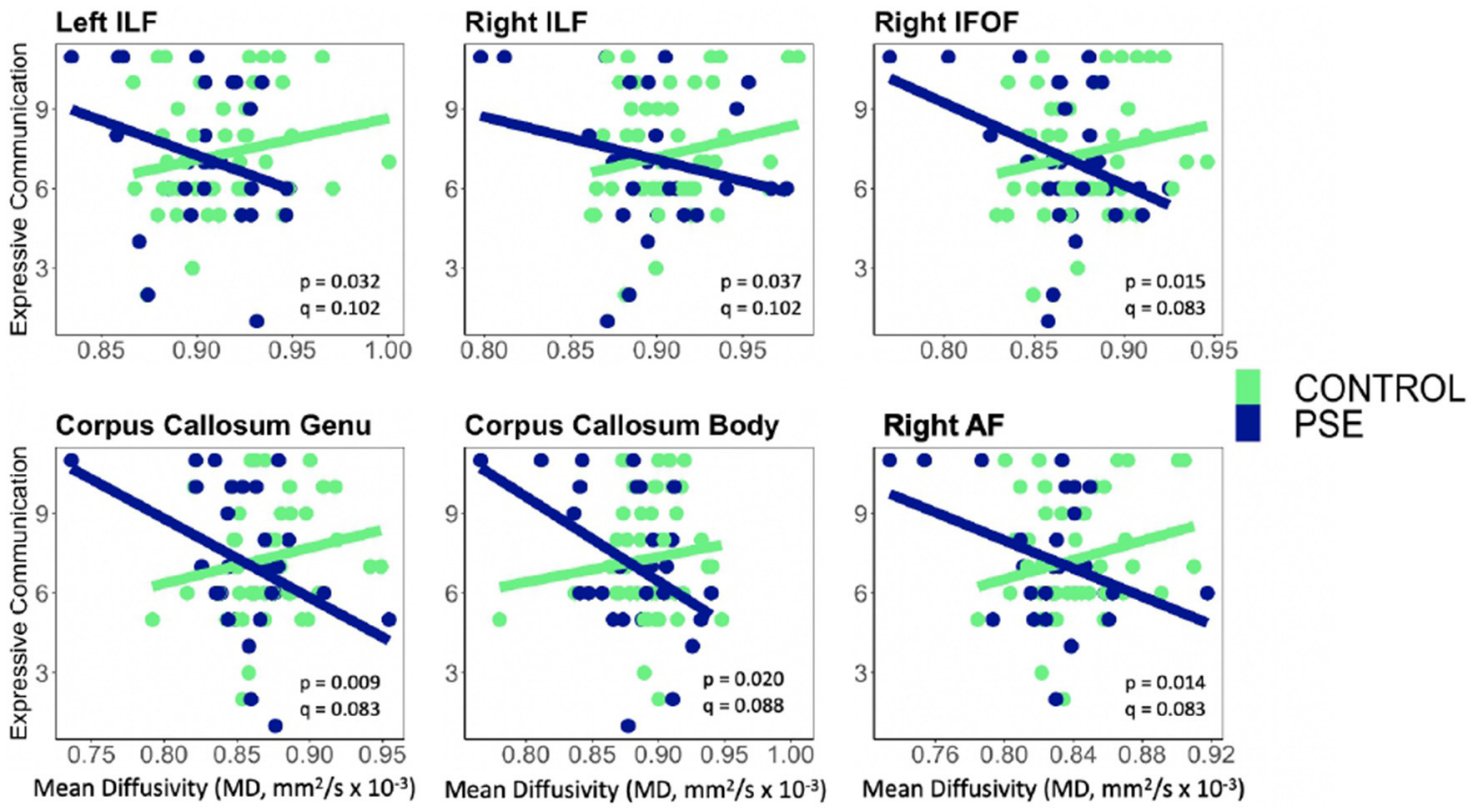
Prenatal Substance Exposure (PSE; defined as prenatal alcohol and/or tobacco exposure)had an interaction with MD in the bilateral ILF, right IFOF, genu and body of the CC, and right AF for Expressive Communication scores. None of these interaction effects survived multiple comparisons correction (*q* > 0.05 for all). PSE group (*n* = 34) can be visualized in blue, and the control group (*n* = 59) is shown in green. ILF, Inferior Longitudinal Fasciculus; IFOF, Inferior Fronto-Occipital Fasciculus; AF, Arcuate Fasciculus.

PSE had both a main effect on language scores and a significant interaction with diffusion metrics in 9 of the 44 models (Tables 2, 3). Expressive Communication and FA models had significant PSE and FA interactions in the left IFOF (*p* = 0.015), and the body of the CC (*p* = 0.035), such that the PSE group had a more positive relationship between FA and Expressive Communication scores than the control group (Figure 4). There was an interaction effect between MD and PSE for Expressive Communication and MD in the bilateral ILF (L = 0.032; R = 0.037), right IFOF (p = 0.015), genu (*p* = 0.009) and body (*p* = 0.020) of the CC, and right AF (*p* = 0.014), such that the PSE group had a more negative relationship between MD and Expressive Communication than controls (Figure 5).

**Table 2:** The main effect of prenatal substance exposure (PSE), as well as the interaction effect of PSE and brain metrics for Expressive Communication models skills in 11 white matter tracts with *p*-values (p), standardized beta (Std. β), and test statistic (t).

**White matter tract**	**Effect**	**Expressive communication**											
		**FA model**						**MD model**					
		**Left**			**Right**			**Left**			**Right**		
		***P*** **(q)**	**Std**. β	***t***	***P*** **(q)**	**Std**. β	***t***	***P*** **(q)**	**Std**. β	***t***	***P*** **(q)**	**Std**. β	***t***
ILF	Age	0.093	0.20	1.70	0.097	0.19	1.68	0.080	0.20	1.78	0.095	0.19	1.69
	Sex	0.972	0.0084	0.04	0.873	0.04	0.16	0.910	0.03	0.11	0.815	0.06	0.23
	Household Income (2)	**0.038 (0.057)**	0.79	2.11	**0.032 (0.057)**	0.81	2.19	**0.040 (0.057)**	0.76	2.09	**0.024 (0.057)**	0.83	2.30
	Brain (FA or MD)	0.815	0.04	0.24	0.684	−0.06	−0.41	0.161	0.20	1.42	0.139	0.24	1.49
	PSE	0.777	0.0043	−0.28	0.406	0.0033	−0.84	**0.032 (0.102)**	−0.0088	2.19	**0.037 (0.102)**	0.0074	2.13
	PSE^*^Brain	0.774	0.07	0.29	0.402	0.21	0.84	**0.032 (0.102)**	−0.50	−2.19	**0.037 (0.102)**	−0.47	−2.12
UF	Age	0.114	0.18	1.60	0.082	0.20	1.76	0.068	0.21	1.85	0.081	0.20	1.77
	Sex	0.975	−0.0074	−0.03	0.918	0.02	0.10	0.987	−0.0037	−0.02	0.973	−0.0076	−0.03
	Household Income	**0.042 (0.057)**	0.75	2.07	**0.040 (0.057)**	0.77	2.10	0.057	0.71	1.94	0.051	0.73	1.98
	Brain (FA or MD)	0.555	−0.09	−0.59	0.804	−0.04	−0.25	0.607	0.08	0.52	0.892	0.02	0.14
	PSE	0.074	0.03	−1.82	0.241	0.05	−1.18	0.104	0.0082	1.65	0.150	0.0012	1.46
	PSE^*^Brain	0.072	0.40	1.83	0.235	0.27	1.20	0.105	−0.37	−1.64	0.150	−0.32	−1.46
IFOF	Age	0.079	0.20	1.78	0.076	0.20	1.80	0.080	0.20	1.78	0.118	0.18	1.58
	Sex	0.884	0.03	0.15	0.935	0.02	0.08	0.943	0.02	0.07	0.836	0.05	0.21
	Household Income	0.201	0.35	1.29	**0.037 (0.057)**	0.77	2.13	**0.047 (0.057)**	0.74	2.02	**0.033 (0.057)**	0.78	2.17
	Brain (FA or MD)	0.122	−0.22	−1.57	0.091	−0.22	−1.71	0.408	0.12	0.83	0.174	0.20	1.37
	PSE	**0.015 (0.083)**	0.01	−2.48	0.077	0.02	−1.79	0.087	−0.04	1.73	**0.015 (0.083)**	−0.03	2.50
	PSE^*^Brain	**0.015 (0.083)**	0.55	2.49	0.075	0.44	1.80	0.087	−0.42	−1.73	**0.015 (0.083)**	−0.55	−2.50
AF	Age	0.269	0.14	1.12	0.097	0.20	1.68	0.085	0.21	1.75	0.117	0.18	1.59
	Sex	0.989	−0.0035	−0.01	0.925	0.02	0.09	0.921	−0.02	−0.10	0.789	0.06	0.27
	Household Income	**0.007 (0.057)**	1.15	2.81	**0.039 (0.057)**	0.80	2.11	0.052	0.79	1.98	**0.047 (0.057)**	0.74	2.02
	Brain (FA or MD)	0.117	0.27	1.59	0.857	−0.03	−0.18	0.637	0.07	0.47	0.121	0.24	1.57
	PSE	0.228	−0.08	1.22	0.200	0.05	−1.29	0.257	−0.12	1.14	**0.014 (0.083)**	−0.0095	2.53
	PSE^*^Brain	0.215	−0.32	−1.25	0.195	0.32	1.31	0.253	−0.30	−1.15	**0.014 (0.083)**	−0.57	−2.52
		**FA Model**						**MD Model**					
		**p**		**Std**. β		**t**		**p**		**Std**. β		**t**	
Genu of the CC	Age	0.076		0.20		1.80		0.066		0.21		1.87	
	Sex	0.977		−0.0070		−0.03		0.716		0.08		0.37	
	Household Income	**0.041 (0.057)**		0.76		2.08		0.074		0.65		1.81	
	Brain (FA or MD)	0.807		0.04		0.25		0.203		0.19		1.28	
	PSE	0.184		−0.0099		−1.34		**0.010 (0.083)**		−0.05		2.66	
	PSE^*^Brain	0.183		0.31		1.34		**0.009 (0.083)**		−0.59		−2.67	
Body of the CC	Age	0.055		0.22		1.95		0.153		0.16		1.44	
	Sex	0.883		−0.04		−0.15		0.788		0.06		0.27	
	Household Income	**0.034 (0.057)**		0.77		2.16		**0.042 (0.057)**		0.74		2.07	
	Brain (FA or MD)	0.867		−0.02		−0.17		0.357		0.15		0.93	
	PSE	**0.036 (0.102)**		0.03		−2.14		**0.020 (0.088)**		−0.07		2.37	
	PSE^*^Brain	**0.035 (0.102)**		0.47		2.15		**0.020 (0.088)**		−0.53		−2.38	
Splenium of the CC	Age	0.105		0.19		1.64		0.088		0.20		1.73	
	Sex	0.996		−0.0014		−0.0056		0.946		−0.02		−0.07	
	Household Income	**0.028 (0.057)**		0.25		2.25		**0.028 (0.057)**		0.84		2.24	
	Brain (FA or MD)	0.711		0.05		0.17		0.476		0.10		0.72	
	PSE	0.869		0.02		0.17		0.672		0.06		0.42	
	PSE^*^Brain	0.872		−0.04		−0.16		0.682		−0.11		−0.41	

P-values before FDR correction; none of the p-values survived FDR correction (q>0.05 for all tracts). Bold lettering statistical significance (p < 0.05).

ILF, inferior longitudinal fasciculus; UF, uncinate fasciculus; IFOF, inferior fronto-occipital fasciculus; AF, arcuate fasciculus; CC, corpus callosum.

**Table 3:** The main effect of prenatal substance exposure (PSE), as well as the interaction effect of PSE and brain metrics for Receptive Communication models skills in 11 white matter tracts with *p*-values (*p*), standardized beta (Std. β), and the *t*-statistic.

**White matter tract**	**Effect**	**Receptive communication**											
		**FA model**						**MD model**					
		**Left**			**Right**			**Left**			**Right**		
		***p*** **(q)**	**Std**. β	***t***	***p*** **(q)**	**Std**. β	***t***	**p (q)**	**Std**. β	***t***	***p (q)***	**Std**. β	***t***
ILF	Age	0.095	0.19	1.69	0.087	0.20	1.73	0.103	0.19	1.65	0.118	0.18	1.58
	Sex	0.163	−0.34	−1.41	0.120	−0.38	−1.57	0.149	−0.35	−1.46	0.189	−0.32	−1.33
	Household income (2)	0.102	0.61	1.65	0.113	0.59	1.60	0.099	0.63	1.67	0.071	0.67	1.83
	Brain (FA or MD)	0.484	0.10	0.70	0.484	0.10	0.70	0.337	0.13	0.97	0.126	0.24	1.55
	PSE	0.562	0.04	0.58	0.278	0.07	1.09	0.380	0.05	0.88	0.194	0.06	1.31
	PSE^*^Brain	0.566	−0.13	−0.58	0.282	−0.26	−1.08	0.385	−0.21	−0.87	0.198	−0.29	−1.30
UF	Age	0.111	0.18	1.61	0.105	0.19	1.64	0.101	0.19	1.66	0.098	0.19	1.68
	Sex	0.159	−0.34	−1.42	0.147	−0.36	−1.47	0.169	−0.34	−1.39	0.155	−0.35	0.39
	Household income (2)	0.123	0.58	1.56	0.121	0.58	1.57	0.113	0.60	1.60	0.120	0.59	1.57
	Brain (FA or MD)	0.738	−0.05	−0.34	0.693	0.06	0.40	0.634	0.07	0.48	0.992	0.0016	0.01
	PSE	0.490	0.05	−0.69	0.966	0.05	0.04	0.744	0.05	0.33	0.749	0.04	0.32
	PSE^*^Brain	0.481	0.16	0.71	0.978	−0.0061	−0.03	0.748	−0.07	−0.32	0.754	−0.07	−0.31
IFOF	Age	0.093	0.20	1.70	0.085	0.20	1.74	0.110	0.18	1.62	0.137	0.17	1.50
	Sex	0.165	−0.34	−1.40	0.169	−0.33	−1.39	0.145	−0.35	−1.47	0.191	−0.31	1.32
	Household income	0.110	0.60	1.62	0.094	0.63	1.69	0.103	0.61	1.65	0.091	0.63	1.71
	Brain (FA or MD)	0.662	−0.06	−0.44	0.583	−0.07	−0.55	0.421	0.11	0.81	0.152	0.21	1.45
	PSE	0.790	0.05	−0.27	0.927	0.06	0.09	0.811	0.08	0.24	0.126	0.04	1.55
	PSE^*^Brain	0.781	0.06	0.28	0.938	−0.02	−0.08	0.821	−0.06	−0.23	0.128	−0.35	−1.54
AF	Age	0.287	0.13	1.07	0.124	0.18	1.56	0.119	0.19	1.58	0.128	0.18	1.54
	Sex	0.175	−0.35	−1.37	0.188	−0.34	−1.33	0.196	−0.33	−1.31	0.176	−0.34	−1.37
	Household income	**0.012 (0.132)**	1.04	2.59	0.100	0.63	1.67	0.071	0.72	1.84	0.098	0.63	1.67
	Brain (FA or MD)	**0.039 (0.669)**	0.35	2.11	0.982	−0.0033	−0.02	0.872	−0.02	−0.16	0.623	0.08	0.48
	PSE	0.236	−0.04	1.20	0.593	0.07	−0.54	0.880	0.02	−0.15	0.634	0.08	0.48
	PSE^*^Brain	0.229	−0.30	−1.21	0.580	0.14	0.56	0.879	0.04	0.15	0.644	−0.11	−0.46
		**FA Model**						**MD Model**					
		**p**		**Std**. β		**t**		**p**		**Std**. β		**t**	
Genu of the CC	Age	0.083		0.20		1.76		0.099		0.19		1.67	
	Sex	0.220		−0.31		−1.24		0.241		−0.29		−1.18	
	Household income	0.124		0.57		1.56		0.126		0.57		1.55	
	Brain (FA or MD)	0.584		−0.08		−0.55		0.210		0.20		1.27	
	PSE	0.226		0.02		−1.22		0.164		0.04		1.40	
	PSE^*^Brain	0.222		0.28		1.23		0.167		−0.31		−1.40	
Body of the CC	Age	0.057		0.22		1.93		0.151		0.16		1.45	
	Sex	0.346		−0.23		−0.95		0.248		−0.28		−1.16	
	Household income	0.135		0.55		1.51		0.082		0.64		1.76	
	Brain (FA or MD)	0.106		−0.24		−1.63		0.062		0.31		1.90	
	PSE	0.068		0.04		−1.85		**0.047 (0.710)**		0.04		2.02	
	PSE^*^Brain	0.066		0.42		1.87		**0.048 (0.700)**		−0.45		−2.01	
Splenium of the CC	Age	0.093		0.20		1.70		0.098		0.19		1.68	
	Sex	0.151		−0.36		−1.45		0.132		−0.37		−1.52	
	Household income	0.109		0.60		1.62		0.075		0.67		1.80	
	Brain (FA or MD)	0.995		0.0010		0.0067		0.239		0.16		1.19	
	PSE	0.793		0.03		−0.26		0.674		0.12		0.42	
	PSE^*^Brain	0.787		0.06		0.27		0.694		−0.11		−0.40	

P-values before FDR correction; none of the p-values survived FDR correction (q>0.05 for all tracts). Bold lettering statistical significance (p < 0.05).

ILF, inferior longitudinal fasciculus; UF, uncinate fasciculus; IFOF, inferior fronto-occipital fasciculus; AF, arcuate fasciculus; CC, corpus callosum.

There were no significant interactions between PSE and FA for Receptive Communication models. There was an interaction effect between PSE and MD for Receptive Communication scores in the body of the CC (*p* = 0.048) similarly to the PSE and MD interactions with Expressive Communication. None of the interactions or main effects survived multiple comparison correction (all *q* > 0.05).

### 3.4 Socioeconomic status

Household income had a positive main effect on Expressive Communication for FA in all tracts, and for MD in all tracts except the bilateral UF, genu of the CC, and left AF (*p* = 0.007–0.038; *q* = 0.057–0.132). There was a main effect of household income in only the left AF for the Receptive Communication models.

#### 3.5.1. *Post hoc* analyses–PAE and PTE

For Expressive Communication, PAE had a main effect and interaction effect with MD in the right IFOF (*p* = 0.017; 0.017, respectively), genu (*p* = 0.005; 0.005) and body (*p* = 0.019; 0.018) of the CC, and right AF (*p* = 0.020; 0.020). For Receptive Communication, PAE had a main effect and interaction with FA in the left AF (*p* = 0.048; 0.043, respectively). PAE had a main effect and interaction with MD for Receptive Communication in the body of the CC (*p* = 0.044, *p* = 0.045, respectively) (Supplementary Tables 1, 2). Similar to our main findings, children with PAE exhibited a more negative relationship between MD and Expressive and Receptive Communication scores, and a more positive relationship between FA and Receptive Communication scores, than controls.

PTE had main and interaction effects for the Receptive Communication and FA model in the body of the CC (*p* = 0.049; 0.046, respectively), and only an interaction effect in the right AF (*p* = 0.047) (Supplementary Tables 3, 4). Following the same trend as the PSE group and PAE independently, participants in the PTE group showed a positive relationship between FA and Receptive Communication scores and controls.

#### 3.5.2. *Post hoc* analyses—cognitive scores

BSID-III Cognitive Scores did not differ between groups. There were no significant interaction effects between PSE and measures of white matter microstructure for the Cognitive Score models, nor were there main effects of PSE on brain metrics.

The previously seen effects in our main analysis became stronger when controlling for Cognitive Score as a covariate. PSE had a main effect and a significant interaction with diffusion metrics in more tracts than in our main analysis. The PSE main effect and PSE and FA interaction effect was significant in the left UF (*p* = 0.005, *p* = 0.004, respectively), left IFOF (*p* = 0.005, *p* = 0.004), genu (*p* = 0.022, *p* = 0.020) and body (*p* = 0.009, *p* = 0.008) of the CC for Expressive Communication scores. There were main effects of PSE and interaction effects of PSE and MD for Expressive Communication in the left UF (*p* = 0.026, *p* = 0.027, respectively), right UF (*p* = 0.017, *p* = 0.017), left IFOF (*p* = 0.013, *p* = 0.014), right IFOF (*p* = 0.003, *p* = 0.003), genu (p < 0.001, p < 0.001) and body (*p* = 0.047, *p* = 0.048) of the CC, and the right AF (*p* = 0.010, *p* = 0.010). PSE had a main effect and interaction with FA for Receptive Communication scores in the genu (*p* = 0.021, *p* = 0.019, respectively) and body (*p* = 0.007, *p* = 0.006) of the CC. PSE had a significant main effect on Receptive Communication scores and MD in the left IFOF (*p* = 0.048).

## 4 Discussion

Here, we show subtle differences in white matter microstructure in children with PSE, as well as a moderating effect of PSE on brain-language relationships, though these did not survive correction for multiple comparisons. In PSE, higher FA and/or lower MD were associated with better language performance in toddlers with PSE, while in controls, this relationship was weak and in the opposite direction. This altered relationship between white matter microstructure and language abilities may reflect early brain differences underlying language challenges that become more evident as children get older.

The PSE group had lower MD in the splenium of the corpus callosum compared to unexposed controls, and a nominally significant difference of MD in the IFOF that did not survive FDR correction. Since MD decreases with age across childhood (Lebel and Deoni, 2018), lower MD could suggest a premature pattern of white matter development. Indeed, altered development patterns have been noted in children with PAE across several prior studies (Kar et al., 2022; Donald et al., 2024). While most studies in older children with PAE report lower FA and/or higher MD compared to unexposed controls (Donald et al., 2015), a few studies have found higher FA and lower MD in brain white matter in 2–7-year-old children with PAE (Kar et al., 2021; Roos et al., 2021; Donald et al., 2024), and even older children with PAE (Lebel et al., 2008; Gómez et al., 2022). In two studies with overlapping datasets but using different methods, similar findings of higher FA and lower MD were observed. Very few studies have looked at white matter microstructure in children with PTE, and results have been somewhat mixed. Jacobsen et al. (2007) found higher FA in the genu of the corpus callosum in adolescents with PTE (many of whom were also current smokers), but young boys prenatally exposed to tobacco and methamphetamine had lower baseline FA and faster decreases in FA compared to unexposed controls when examined longitudinally (Chang et al., 2016). Further research, particularly longitudinal research, is needed to better understand the trajectories of FA and MD in children with PSE.

There were no significant group differences in Expressive or Receptive Communication scores. The existing literature shows conflicting results, especially in younger children, with some studies reporting worse expressive and receptive communication performance in young children with PAE (Fried and Watkinson, 1988, 1990; McGee et al., 2009; Davies et al., 2011), and others finding no language differences in the toddler years (Greene et al., 1990; Kaplan-Estrin et al., 1999; Hendricks et al., 2020). In general, the studies that found no language differences included participants with low-moderate PAE (Greene et al., 1990; Kaplan-Estrin et al., 1999), while studies of children with higher PAE reported worse language skills (McGee et al., 2009). This perhaps reflects a dose-dependent relationship, with language impairments being more evident in children with worse exposures. Our sample had a wide range of PAE, and moderate-to-high levels of PTE, though we did not have enough participants at each exposure level to test a dose-dependent relationship. Future studies are needed to examine the role of the amount of PSE on language difficulties in toddlers.

Higher FA and lower MD were associated with better language skills in the PSE group, while the control group showed weaker and opposite relationships. In typically developing older children and adults, higher FA and lower MD in several white matter tracts are consistently associated with better language performance (Ivanova et al., 2016; Mürner-Lavanchy et al., 2018), which is consistent with the relationships observed here in children with PSE. Indeed, subtle structural alterations in white matter may underlie language performance in the children with PSE and lead to the associations observed here. However, in contrast to our results, Gómez et al. (2022) reported a negative association between a language score component closely associated with speeded naming, and FA, in the genu of the corpus callosum in older children with PAE. This may reflect a complex role of age in these relationships, which is in line with altered development trajectories in children with PAE (Kar et al., 2022; Donald et al., 2024). Young children with PAE tend to have slower decreases of MD over time in several white matter tracts compared to unexposed controls, which may indicate reduced brain plasticity and/or a prolonged period of brain development, both have implications for cognitive outcomes and language abilities (Lebel et al., 2012; Kar et al., 2022; Donald et al., 2024). Longitudinal studies will help better elucidate these brain-language relationships across age.

In this study, household income was significantly associated with language scores, which is in line with prior studies of unexposed children (Noble et al., 2005; Schwab and Lew-Williams, 2016). Hemingway et al. (2020) found that PAE explained a higher proportion of variance in brain structure and function compared to other risk factors including socioeconomic status. Other studies have found attenuated or absent associations between current socioeconomic status (caregiver's educational attainment, occupation, and household income) and brain metrics in children with PAE (McLachlan et al., 2020; Uban et al., 2020). This may reflect the pre- to post-natal changes in socioeconomic factors for these children, who were primarily no longer living with their birth families. All participants in this study lived with their biological mother and came from households where incomes were lower than the mean annual household income for Statistics South Africa (2017) (R138,168/y, equivalent to ~$7,500/y USD; Statistics South Africa, 2017). Most previous studies investigating the effects of PAE and PTE were conducted on individuals coming from families with higher incomes, which may influence developmental outcomes of PAE (Uban et al., 2020). The geographical region and group of people that were investigated in the current study is one of the most impacted populations affected by PSE, making it an important cohort to study. It is possible that the influence of household income on brain and language outcomes varies at different levels of PSE and income, and/or when children remain in the same families (Hendricks et al., 2020).

We observed similar trends for the effects of PAE and PTE independently, as we did for the effects of PSE altogether. This suggests that at these levels, and in this population, PTE has similar effects on the brain-language relationship as PAE. It is worth noting, however, that many previous studies of PAE included participants with co-occurring PTE and/or other stubstances (Hendricks et al., 2019; Roos et al., 2021; Kar et al., 2022; Donald et al., 2024). Future, larger studies may be able to examine the interaction between the two exposures to provide more insight into combined or separate prenatal exposures.

Our results appear to reflect language abilities specifically, rather than more general intellectual ability, as the supplementary analysis for cognitive scores showed no effects. Furthermore, when adding cognitive scores as a covariate in our language models, we saw the same trends as in our main analysis but with stronger relationships.

One limitation of this study is the small sample size, preventing us from examining interactions between alcohol and tobacco exposure. This also limited our ability to detect significant moderation effects, and none of these survived multiple comparison corrections.

In conclusion, we show that PSE appears to moderate the brain-language relationship in early life. While language deficits have not yet emerged, the altered brain-language relationship in the toddler years could be the basis of language challenges in older children with PSE. Substance-exposed children could face different challenges than unexposed children as they begin to engage in more complicated language activities including learning to read. Furthermore, this highlights the importance of developing early interventions for children with PSE to mitigate the development of language challenges.

## Data Availability

The data analyzed in this study is subject to the following licenses/restrictions: Data will be made available on request. Requests to access these datasets should be directed to kirsty.donald@uct.ac.za.
