# Supplementary material for: Prenatal tobacco and alcohol exposure, white matter microstructure, and early language skills in toddlers from a South African birth cohort
Source: Front Integr Neurosci. 2024 Sep 2;18:1438888. doi: 10.3389/fnint.2024.1438888 (PMC11402807; doi:10.3389/fnint.2024.1438888)
Supplement: Supplementary file 1 [file Table_1.docx]

Supplementary Material

# Supplementary Figures and Tables

Supplementary Table 1: The main effect of prenatal alcohol exposure (PAE), as well as the interaction effect of PAE on the brain-language relationship for Expressive Communication skills in 11 white matter tracts with p-values (p) and standardized beta (Std. β).

| White Matter Tract | Effect | Expressive Communication | | | | | | | | |
| --- | --- | --- | --- | --- | --- | --- | --- | --- | --- | --- |
|  |  | FA Model | | | | MD Model | | | | |
|  |  | Left | | Right | | Left | | Right | | |
|  |  | p | Std. β | p | Std. β | p | Std. β | p | | Std. β |
| ILF | PAE | 0.619 | -0.0097 | 0.995 | -0.01 | 0.081 | -0.05 | 0.056 | | -0.05 |
|  | PAE*Brain | 0.615 | -0.13 | 0.997 | 0.0010 | 0.080 | -0.45 | 0.056 | | -0.46 |
| UF | PAE | 0.150 | -0.0035 | 0.579 | -0.02 | 0.118 | -0.05 | 0.092 | | -0.08 |
|  | PAE*Brain | 0.150 | 0.35 | 0.582 | 0.14 | 0.117 | -0.39 | 0.090 | | -0.40 |
| IFOF | PAE | 0.056 | -0.02 | 0.253 | -0.02 | 0.126 | -0.10 | **0.017** | | -0.10 |
|  | PAE*Brain | 0.056 | 0.47 | 0.254 | 0.32 | 0.125 | -0.42 | **0.017** | | -0.59 |
| AF | PAE | 0.175 | -0.23 | 0.325 | -0.06 | 0.307 | -0.28 | **0.020** | | -0.08 |
|  | PAE*Brain | 0.153 | -0.23 | 0.326 | 0.30 | 0.294 | -0.30 | **0.20** | | -0.57 |
|  | |  | | | | MD Model | | | | |
|  |  | p | | Std. β | | p | | | Std. β | |
| Genu of the CC | PAE | 0.258 | | -0.07 | | **0.005** | | | -0.18 | |
|  | PAE*Brain | 0.261 | | 0.28 | | **0.005** | | | -0.69 | |
| Body of the CC | PAE | 0.052 | | -0.04 | | **0.019** | | | -0.16 | |
|  | PAE*Brain | 0.052 | | 0.50 | | **0.018** | | | -0.57 | |
| Splenium of the CC | PAE | 0.639 | | -0.0096 | | 0.699 | | | 0.0053 | |
|  | PAE*Brain | 0.635 | | -0.13 | | 0.703 | | | -0.11 | |

Note. P-values before FDR correction; bold lettering indicates statistical significance (p<0.05)

Supplementary Table 2: The main effect of prenatal alcohol exposure (PAE), as well as the moderation effect of PAE on the brain-language relationship for Receptive Communication skills in 11 white matter tracts with p-values (p) and standardized beta (Std. β).

| White Matter Tract | Effect | Receptive Communication | | | | | | | | |
| --- | --- | --- | --- | --- | --- | --- | --- | --- | --- | --- |
|  |  | FA Model | | | | MD Model | | | | |
|  |  | Left | | Right | | Left | | Right | | |
|  |  | p | Std. β | p | Std. β | p | Std. β | p | | Std. β |
| ILF | PAE | 0.163 | 0.06 | 0.079 | 0.08 | 0.614 | 0.02 | 0.427 | | 0.05 |
|  | PAE*Brain | 0.164 | -0.35 | 0.079 | -0.48 | 0.617 | -0.13 | 0.432 | | -0.19 |
| UF | PAE | 0.867 | 0.0009 | 0.478 | 0.0076 | 0.958 | 0.02 | 0.892 | | 0.0095 |
|  | PAE*Brain | 0.867 | -0.04 | 0.480 | -0.18 | 0.956 | 0.01 | 0.894 | | -0.03 |
| IFOF | PAE | 0.568 | 0.02 | 0.440 | 0.02 | 0.894 | 0.07 | 0.341 | | 0.02 |
|  | PAE*Brain | 0.569 | -0.14 | 0.442 | -0.22 | 0.888 | 0.04 | 0.344 | | -0.23 |
| AF | PAE | **0.048*** | -0.13 | 0.616 | 0.07 | 0.293 | -0.03 | 0.776 | | 0.05 |
|  | PAE*Brain | **0.043*** | -0.13 | 0.624 | -0.15 | 0.298 | 0.30 | 0.783 | | -0.07 |
|  | |  | | | | MD Model | | | | |
|  |  | p | | Std. β | | p | | | Std. β | |
| Genu of the CC | PAE | 0.534 | | 0.0055 | | 0.173 | | | -0.0096 | |
|  | PAE*Brain | 0.531 | | 0.16 | | 0.174 | | | -0.33 | |
| Body of the CC | PAE | 0.304 | | 0.02 | | **0.044** | | | -0.02 | |
|  | PAE*Brain | 0.300 | | 0.28 | | **0.045** | | | -0.49 | |
| Splenium of the CC | PAE | 0.445 | | 0.07 | | 0.635 | | | 0.07 | |
|  | PAE*Brain | 0.448 | | -0.21 | | 0.648 | | | -0.14 | |

Note. P-values before FDR correction; bold lettering indicates statistical significance (p<0.05)

Supplementary Table 3: The main effect of prenatal tobacco exposure (PTE), as well as the interaction effect of PTE on the brain-language relationship for Expressive Communication skills in 11 white matter tracts with p-values (p) and standardized beta (Std. β).

| White Matter Tract | Effect | Expressive Communication | | | | | | | | |
| --- | --- | --- | --- | --- | --- | --- | --- | --- | --- | --- |
|  |  | FA Model | | | | MD Model | | | | |
|  |  | Left | | Right | | Left | | Right | | |
|  |  | p | Std. β | p | Std. β | p | Std. β | p | | Std. β |
| ILF | PTE | 0.090 | -0.30 | 0.127 | -0.0008 | 0.146 | 0.07 | 0.245 | | 0.13 |
|  | PTE*Brain | 0.086 | 0.73 | 0.119 | 0.63 | 0.146 | -0.60 | 0.254 | | -0.41 |
| UF | PTE | 0.209 | 0.03 | 0.156 | 0.19 | 0.454 | 0.18 | 0.984 | | 0.21 |
|  | PTE*Brain | 0.197 | 0.47 | 0.143 | 0.48 | 0.464 | -0.27 | 0.971 | | 0.01 |
| IFOF | PTE | 0.145 | -0.13 | 0.227 | 0.02 | 0.288 | 0.13 | 0.275 | | 0.15 |
|  | PTE*Brain | 0.137 | 0.68 | 0.215 | 0.61 | 0.298 | -0.45 | 0.284 | | -0.43 |
| AF | PTE | 0.351 | 0.67 | 0.246 | 0.50 | 0.774 | 0.80 | 0.296 | | -0.10 |
|  | PTE*Brain | 0.405 | -0.34 | 0.231 | 0.050 | 0.744 | 0.20 | 0.306 | | -0.50 |
|  | |  | | | | MD Model | | | | |
|  |  | p | | Std. b | | p | | | Std. b | |
| Genu of the CC | PTE | 0.537 | | 0.13 | | 0.796 | | | 0.19 | |
|  | PTE*Brain | 0.523 | | 0.28 | | 0.809 | | | -0.10 | |
| Body of the CC | PTE | 0.360 | | 0.18 | | 0.468 | | | 0.12 | |
|  | PTE*Brain | 0.340 | | 0.31 | | 0.477 | | | -0.34 | |
| Splenium of the CC | PTE | 0.886 | | 0.19 | | 0.879 | | | 0.26 | |
|  | PTE*Brain | 0.904 | | -0.04 | | 0.896 | | | -0.09 | |
|  |  |  |  |  |  |  |  |  |  |  |

Note. P-values before FDR correction; bold lettering indicates statistical significance (p<0.05)

Supplementary Table 4: The main effect of prenatal tobacco exposure (PTE), as well as the interaction effect of PTE on the brain-language relationship for Receptive Communication skills in 11 white matter tracts with p-values (p) and standardized beta (Std. β).

| White Matter Tract | Effect | Receptive Communication | | | | | | | |
| --- | --- | --- | --- | --- | --- | --- | --- | --- | --- |
|  |  | FA Model | | | | MD Model | | | |
|  |  | Left | | Right | | Left | | Right | |
|  |  | p | Std. β | p | Std. β | p | Std. β | p | Std. β |
| ILF | PTE | 0.267 | -0.18 | 0.827 | 0.07 | 0.362 | 0.01 | 0.113 | -0.0071 |
|  | PTE*Brain | 0.268 | 0.41 | 0.815 | 0.09 | 0.370 | -0.39 | 0.116 | -0.58 |
| UF | PTE | 0.055 | -0.05 | 0.493 | 0.16 | 0.333 | 0.06 | 0.530 | 0.10 |
|  | PTE*Brain | 0.052 | 0.65 | 0.472 | 0.23 | 0.338 | -0.35 | 0.536 | -0.24 |
| IFOF | PTE | 0.207 | -0.11 | 0.619 | 0.06 | 0.381 | 0.06 | 0.061 | -0.03 |
|  | PTE*Brain | 0.204 | 0.48 | 0.607 | 0.21 | 0.390 | -0.38 | 0.063 | -0.72 |
| AF | PTE | 0.701 | 0.04 | 0.051 | 0.28 | 0.108 | -0.22 | 0.533 | 0.09 |
|  | PTE*Brain | 0.695 | -0.15 | **0.047** | 0.76 | 0.113 | -0.96 | 0.542 | -0.27 |
|  | |  | | | | MD Model | | | |
|  |  | p | | Std. b | | p | | Std. b | |
| Genu of the CC | PTE | 0.092 | | -0.09 | | 0.674 | | 0.10 | |
|  | PTE*Brain | 0.089 | | 0.75 | | 0.683 | | -0.17 | |
| Body of the CC | PTE | **0.049** | | 0.05 | | 0.731 | | 0.14 | |
|  | PTE*Brain | **0.046** | | 0.63 | | 0.740 | | -0.15 | |
| Splenium of the CC | PTE | 0.124 | | -0.06 | | 0.819 | | 0.26 | |
|  | PTE*Brain | 0.121 | | 0.57 | | 0.841 | | -0.13 | |

Note. P-values before FDR correction; bold lettering indicates statistical significance (p<0.05)
